# Sleep disturbances and mental strain in university students: results from an online survey in Luxembourg and Germany

**DOI:** 10.1186/s13033-017-0131-9

**Published:** 2017-03-29

**Authors:** Angelika A. Schlarb, Merle Claßen, Julia Grünwald, Claus Vögele

**Affiliations:** 1 Department of Psychology and Sports, Faculty for Psychology and Sports, Bielefeld University, POP 10 01 31, 33501 Bielefeld, Germany; 2 Department of Psychology, Faculty of Science, University of Tübingen, Schleichstrasse 4, 72076 Tübingen, Germany; 3 Research Unit INSIDE, Institute for Health and Behaviour, University of Luxembourg, 2, Avenue de l’Université, L-4365 Esch-Sur-Alzette, Luxembourg

**Keywords:** Sleep, Depression, Stress, European students, Mental health

## Abstract

**Objectives:**

This study examined the prevalence of sleep disturbances and mental strain in students from two European countries, Luxembourg and Germany.

**Methods:**

A total of 2831 students took part in an online survey, with 2777 students from Germany and 184 students from Luxembourg. Sleep disturbances were assessed with the Pittsburgh Sleep Quality Index and the Epworth Sleepiness Scale, and aspects of mental strain using the Patient Health Questionnaire, the Social-Interactive-Anxiety Scale, the self-efficacy questionnaire and the test anxiety questionnaire. In addition, we also assessed students’ chronotypes.

**Results:**

Across the whole sample mean scores on the sleep questionnaires were above the cut-off for clinically relevant sleep problems, indicating an increased prevalence of sleep disturbances in students from both countries. Sleep quality was impaired in 42.8%, and 17.9% showed clinically relevant scores. Overall 25.5% reported elevated depression and 13.3% social phobia symptoms, while 45% indicated elevated stress levels. Sleep quality, daytime sleepiness, chronotype, depression scores, stress levels, test anxiety, and self-efficacy differed significantly between men and women, but there were no differences between countries.

**Conclusions:**

Sleep disturbances and mental strain in students are common, with the current results replicating previous findings. Students from Luxembourg and Germany are affected equally.

## Background

Going to university is associated with changes in lifestyle, with increasing autonomy in personal life. Students’ social environment changes, as many move out from their parents’ homes into shared or single flats [1]. Highly variable starting times for university lectures and seminars in the morning often lead to changing sleeping patterns [2]. University students are at high risk for developing sleep problems, with symptoms such as difficulties falling asleep, frequent night awakenings, nightmares [3] and daytime impairments [2]. Risk factors, e.g. rising late, short sleep durations and non-restorative sleep concern especially university students. Many report bedtimes differing more than 2 h on weekdays and weekends [4]. Nevertheless, previous findings are inconsistent in terms of reported percentages of serious sleep disorders in university students and in the general population [5]. The highest prevalence of poor sleep quality and occasional sleep disturbances (73%) was reported for US university students [6]. Current estimates of the percentage of university students all over the world fulfilling diagnostic criteria for insomnia range from 9.4–13.1% [2, 7, 8]. Sleep disorders, poor sleep quality and excessive daytime sleepiness is associated with lower academic motivation and lower self-efficacy [9]. In university students without depression, poor sleep quality has been linked to lower academic performance [9]. Sleep disturbances often occur during high-stress periods, with stress and sleep disturbances increasing over a 4-year period in Canadian university students [10]. Concerning the widespread influences of disturbed sleep on learning and other cognitive functions in students, an experimental study showed that students are not aware of the impact of sleep restriction on their academic performance [11]. They overrated their academic performance after total sleep deprivation. This could result in poorer academic performance in many European universities without the students knowing why. In addition, Daley and colleagues showed that insomnia was associated with less work productivity, increased health care utilization and work absenteeism [12]. Moreover, insufficient sleep is also a risk factor for burnout [13].

In addition to sleep and insomnia, other factors have been shown to affect university students’ well-being. For example, chronotype is associated with stress responses [14]: evening-types are more vulnerable to stress, and show impaired academic motivation. This association, is mediated by daytime sleepiness. An association between chronotype and academic performance has also been demonstrated in a Turkish sample of university students [15]. Evening-types are assumed to be at an academic disadvantage with exams and lectures mostly scheduled in the mornings. In contrast, higher self-efficacy is associated with better academic performance, self-regulation, mental health and fewer sleep disturbances [16].

Thees et al. [17] investigated self-reported health in German university students. The majority of participants reported elevated stress levels after the change from the previous higher education system to the Bologna system. In their study, prevalence rates for different impairments were assessed for a range of physical complaints such as headaches, stomach aches, sleeping problems, back pain and muscular tension. 44.6% reported regular muscular tension, 30.6% back pain, 9.3% tinnitus and 20% sleep disturbances. In total, about one quarter reported ill health. In a sample of 1130 university students at a German university, 22.7% fulfilled criteria for a mental disorder excluding alcohol syndrome [18]. Most prevalent among university students were depression (14.1%) and somatoform syndrome (9.1%). Nevertheless, sleep quality was not included in Bailer and colleague’s study. In another large study concerning health of college students and non-college attending young adults, 45.8% of college students had a mental disorder [19]. Again, sleep disorders were not considered in the analysis. Alcohol syndrome was the most common disorder, 20.3% of all college students reported an alcohol-related disorder [19]. These were the only syndromes more frequent in college students than in their non-college-attending peers. Mood disorders, anxiety disorders, and personality disorders were less prevalent in college students than in their non-college peers. Various studies have demonstrated, that mental health and insomnia are often related to lower academic grades [8, 20], and it is estimated that 3.2–11.4% of college non-completion variance is explained by mental disorders [8].

Gender differences in psychological distress have also been found in European university students. Women are significantly more often affected (OR = 1.8) by a mental disorder than men, excluding alcohol syndrome [18]. In a large Turkish sample, female university students indicated significantly higher stress and anxiety scores compared to male students [21, 22]. Furthermore, higher test anxiety is significantly related to lower performance in tests, and female university students are significantly more often impaired than male students [23].

Various components of mental strain interact. Sleep problems co-occur regularly with various mental health impairments, as depression, anxiety disorders, and substance abuse in college students [8]. Depression, anxiety, and sleep problems often co-vary and influence each other [24, 25]. Chronotypes correspond differently to stress, which influences sleep quality and quality of life as a result [14, 26]. A high impact of self-efficacy on depressive and anxiety symptoms, as well as sleep quality, was reported [14, 27]. Even after controlling for trait anxiety, self-efficacy influenced internalizing symptoms significantly [27].

The aim of this study, therefore, was to evaluate sleep disturbances, mental strain and self-efficacy in two samples of university students from two different German speaking EU countries, and relate these to mental strain. We wanted to examine [1] if German and Luxembourgish students report an equal level of sleep disturbances, as well as equal sleep duration, sleep-onset latency, daytime sleepiness, and amount of chronotypes due to cultural similarities and largely similar educational systems. Hence, [2] if depression and other mental strains are on the same level in both countries. Thirdly, [3] if gender differences concerning sleep quality, chronotype, depression, measures of anxiety, and self-efficacy are equal as shown in previous studies.

## Methods

### Procedure

An online survey was conducted in 19 universities, while the majority of the participants studied at two German universities (91.7%) and at the University of Luxembourg (6.5%). University students were invited via all accessible e-mail circulators to all university students from Tübingen (Germany), Koblenz-Landau (Germany), and Luxembourg. Participants filled out the questionnaires voluntarily and were given information about goal and content of the study prior to participation. Prior to being able to access the survey questions, volunteers were requested to give their written, informed consent. Participants were able to exit the survey at any time if they so wished. The goal was to recruit an approximately similar percentage of university students of each country. The study design was approved by the ethics committee of Bielefeld University.

### Study sample

The total sample consisted of 2831 students (age M = 23.71; SD = 3.72; range 17–59), of which 762 were men (26.7%) and 2095 were women (73.3%). On average the students studied 5.83 semesters (SD = 3.70; range 1–30) with German students reporting a significantly higher number of semester (t (2816) = 5.425; p = .000). A breakdown according to country is provided in Table 1. Overall, the response rate in German universities (Tübingen and Landau) was 6 whereas 3% of Luxembourgish students took part. In detail, 73% of the present sample were from University in Tübingen, 18.7% from University Koblenz-Landau, 6.5% from Luxembourg University and 1.8% from other universities throughout Germany (e.g. Würzburg University, Stuttgart University; 16 different universities in total but only few participants each).

**Table 1:** Gender and age in total and for all diagnostic groups

Group	Sum	Gender		Age	Semesters
	N (%)	Male	Female	M (SD)	M (SD)
Germany	2646 (90.27%)	697 (26.3%)	1949 (73.7%)	23.76 (3.70)	5.93 (3.75)
Luxembourg	184 (9.73%)	59 (32.1%)	125 (67.9%)	23.07 (3.97)	4.42 (2.65)
Total	2830 (100%)	756 (26.71%)	2074 (73.29%)	23.71 (3.72)	5.83 (3.70)

*M* mean; *SD* standard deviation

### Diagnostic measures

The diagnostic measures included the Pittsburgh Sleep Quality Index (PSQI, 28), which assesses retrospectively the sleep quality over the previous four weeks. A total of 18 items (ranging from 0–3) sum up to seven different sub-scales (sleep quality, sleep-onset latency, sleep duration, habitual sleep efficiency, sleep disturbance, use of sleep medication, daytime dysfunction). The total score is calculated by summing all sub-scale scores with a cut-off score of >5 indicating “bad sleepers” in comparison to “good sleepers” (≤5). A total score >10 indicated a severe sleep problem or sleep disorder. Reliabilities for the PSQI sum score between 0.82 and 0.89 and good specificity and sensitivity and were reported [28].

The Epworth Sleepiness Scale (ESS) was used as a short questionnaire to assess daytime sleepiness [29]. The ESS assesses retrospectively the probability to fall asleep in eight everyday situations by means of a scale ranged from 0 (=never fall asleep) to 3 (=high probability to fall asleep). The total sum score ranges from 0 to 24. According to Bloch and colleagues [30] we rated sum scores ≥10 as clinically significant elevated. The German validated version revealed good reliability (α = 0.83) and validity [30].

To assess chronotype the Morningness-Eveningness-Questionnaire (German version; dMEQ) was used [31]. Questions about preferred time for getting up, going to sleep, practicing sports or other activities sum up into a total score. Within 19 questions preferred daytimes for activities are asked and a sum score is built. Scores below 42 suggest an evening type, scores above 58 identify morning types and lying between are neutral types. Authors reported good reliability and significant correlations to other chronotype questionnaires and melatonin measurements [31].

The PHQ-D (Patient Health Questionnaire, German version) was implemented as a screening instrument to assess potential symptoms of depression and stress [32] with the respective modules for depressive disorders and stress, each including nine items. For both modules, the following categorization was used: (0–4) minimal symptomatology, [5–9] mild symptomatology, [10–14] moderate symptomatology, [15–20] severe symptomatology. Internal consistency of the continuous subscales was α = 0.88 and classification according to diagnostic criteria was excellent [33].

Social phobia was assessed with the SIAS questionnaire (social-interaction-anxiety scale), a self-assessment instrument including 20 items with a five-point scale ranging from 0 = not at all to 4 = at all [34]. A cut-off of ≥34 indicates social phobia. Internal consistency of α = 0.86 for patients with social phobia and α = 0.90 for control subjects was reported [34].

To measure test anxiety we used the test anxiety questionnaire (“Prüfungsangstfragebogen”, PAF), which assesses specific aspects of test anxiety in school and university students. The questionnaire consists of 20 items with four scales (nervousness, concerns, interference and lack of confidence), answered on a 4-point Likert scale. A summary score is calculated and compared to a university students’ population score (age M = 25 years; SD = 5) [35]. Scores higher than 53 are deemed to be clinically relevant. The sum score revealed good reliability (α = 0.88) and validity.

Perceived self-efficacy was assessed using the self-efficacy questionnaire (SWE; [36]), which consists of 10 items (range from [1] disagree, [2] agree hardly, [3] agree rather to [4] agree completely). A sum score is calculated by adding up all responses (score between 10 and 40). A sum score, lower than 23, indicates clinically relevant low self-efficacy. Reliability between α = 0.80 and α = 0.90 for the SWE sum score were reported [37].

### Statistical analysis

For statistical analyses, the Statistical Package for Social Science (SPSS, version 22.0) was used. Normal distribution was tested with the Kolmogorov–Smirnov test for PSQI subscales, and comparisons were carried out using Mann–Whitney-U tests. We assumed normal distribution due to the large sample size in total scores of all questionnaires [38]. Differences between sub-groups (i.e. country, sex) were investigated using MANOVA, followed by univariate comparisons, if significant. Assumptions for MANOVA were assessed: test of equality of covariance matrices was not significant. Pillai’s trace is reported due to unequal sample sizes. Separate multivariate linear regression was calculated to estimate the effect of continuous variables on various outcome variables when an inclusion into MANOVA was not possible. The level of significance was set at α ≤ .05.

## Results

### Sleep

The PSQI was used to examine sleep quality of the last 4 weeks. On average, the total score was above the cut-off for good sleepers suggesting that self-reported sleep quality was impaired (M = 7.22; SD = 3.70). More specifically, 42.8% had impaired sleep quality with a PSQI total score above 5, and 17.9% had severe sleep problems according to the PSQI (>cut-off 10). In both countries, an equal proportion of students reported sleep disturbances or sleep disorders according to the PSQI (χ^2^ = 2.914, p = .233) (see Table 2). Women and men differed significantly concerning the proportion of bad sleep quality and severe sleep problems (χ^2^ = 7.773, p = .021). 41.8% women reported bad sleep quality compared to 46.0% men and 19.1% women had severe sleep problems compared to 14.9% men.

**Table 2:** Percentage of sleep disturbances and sleep disorders in German and Luxembourgish students

Sleep quality	All students (%)	Germany (%)	Luxembourg (%)
Good sleep quality (PSQI ≤ 5)	39.3	39.4	34.8
Impaired sleep (PSQI 6–10)	42.8	42.9	42.9
Severe sleep problem (PSQI > 10)	17.9	17.7	22.3

Due to the non-normal distribution of some PSQI subscales, Mann–Whitney-U-tests were carried out to test for differences between countries, which showed no statistically significant effects (see Appendix Table 8). MANOVA results indicated no differences in overall sleep quality between students from both countries (see Appendix Table 9), but gender showed a main effect (see Table 3). Female participants showed worse sleep than men in the whole sample, as well as in Germany and Luxembourg, separately (no interaction effect, see Table 5). Nevertheless, PSQI subscales differed significantly between gender for PSQI subscale sleep disturbances and daytime sleepiness (see Table 4).

**Table 3:** Gender differences in mental health

Variable	Female university students M (SD)	Male university students M (SD)	Significance^a^
PSQI—sleep quality for all students	7.33 (3.77)	6.94 (3.47)	F (1, 2593) = 6.17; p = .013*
ESS—daytime sleepi-ness for all students	8.59 (3.75)	7.63 (3.59)	F (1, 2573) = 33.73; p = .000**
MEQ—chronotype for all students	46.85 (9.38)	44.22 (9.82)	F (1, 2781) = 38.34; p = .000**
PHQ-9—depression for all students	7.54 (4.80)	6.56 (4.81)	F (1, 2632) = 21.02; p = .000**
PHQ—stress for all students	5.39 (3.22)	4.62 (2.84)	F (1, 2613) = 52.23; p = .000**
SIAS—social phobia for all students	23.62 (10.59)	23.96 (10.82)	F (1, 2573) = 0.47; p = .495
PAF—test-anxiety for all students	44.90 (6.71)	43.71 (9.82)	F (1, 2693) = 19.12; p = .000**
SWE—self-efficacy for all students	28.19 (4.76)	29.18 (5.21)	F (1, 2593) = 21.99; p = .000**

^a^According to MANOVA; * p ≤ .05; ** p < .01

**Table 4:** Means, standard deviations and significance level for the components of the PSQI

	Scale	Women		Men		Significance level
		Mdn	M (SD)	Mdn	M (SD)	
(1)	Sleep quality	1		1		U = 812,926; z = 1.817; p = .069
(2)	Sleep-onset latency In minutes	2	25.48 (28.53)	2	22.52 (21.27)	U = 797,021, z = 1.285; p = .199
(3)	Sleep duration In hours	0	7.10 (1.10)	0	7.02 (1.07)	U = 773,050; z = -.233; p = .816
(4)	Habitual sleep efficiency In percent	0	87.35 (10.32)	0	88.81 (9.65)	U = 792,965; z = 1.190; p = .234
(5)	Sleep disturbance	1		1		U = 880,629; z = 7.144; p = .000*
(6)	Use of sleep medication	0		0		U = 786,964; z = .432; p = .666
(7)	Daytime dysfunction	1		1		U = 812,662; z = 2.510; p = .012*

*Mdn* median; *M* mean; *SD* standard deviation; * p ≤ 0.05

**Fig. 1:**
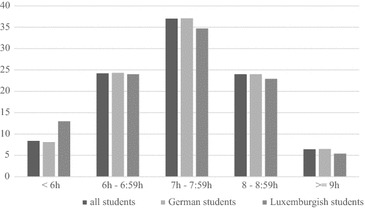
Sleep duration in percent in German and Luxembourgish university students

University students reported to sleep from 3 to 12 h per night. Most of the students slept between 7 and 7:59 h (Mdn = 7). In hours women reported significantly longer sleep durations (Mdn_women_ = 7; Mdn_men_ = 7; U = 815,341; z = 2.055; p = .040) (Fig. 1).

In the whole sample, 18.8% reported sleep-onset latency of more than 30 min, with a range of zero minutes up to 6 h (Mdn = 15.0 min). In German students 18.4% reported sleep-onset latencies of approximately 30 min (Md = 15.0 min, see Table 8). In Luxembourg, students regularly fell asleep after 1 min up to 3 h (Md = 20.0 min). 24.6% of Luxembourgish students reported sleep-onset latency of over 30 min. This difference was significant (χ^2^ = 4.292, p = .038). Women reported significantly longer sleep-onset latency in minutes (Mdn_women_ = 15; Mdn_men_ = 15; U = 822,420; z = 2.173; p = .030). A sleep-onset latency longer than 30 min is significantly more often stated by women (19.7%) compared to men (16.3%; χ^2^ = 4.258, p = .039).

Nightmares occurred less than once per week (M = .76; SD = .85). 34.5% of German university students and 25.5% of Luxembourgish students reported nightmares less than once a week. In Germany, 14.5% had nightmares once or twice a week and 4.2% thrice or more often, whereas in Luxembourg 18.5% experienced nightmares once or twice a week and 4.9% more than that. 35.8% women (28.8% men) reported nightmares less than once a week, 16.8% women (9.1% men) had nightmares once or twice a week and 5.2% women (1.7% men) more often. Hence women experience nightmares significantly more often (χ^2^ = 86.301, p = .000).

Using MANOVA to investigate effects of gender and country on mental health, a main effect for gender occurred for sleep quality, daytime sleepiness, chronotype, depression, stress, social phobia, test anxiety, and self-efficacy (see Table 5).

**Table 5:** Results of MANOVA testing effects of country and gender on sleep quality, daytime sleepiness, chronotype, depression, stress, social phobia, test anxiety and self-efficacy

Variable	Pillai’s trace		Significance	Effect size
Country	0.003	F (8, 2471) = 0.91	p = .553	η^2^ = .003
Gender	0.029	F (8, 2471) = 9.29	p = .000*	η^2^ = .029
Country* gender	0.004	F (8, 2471) = 1.39	p = .265	η^2^ = .004

* p < 0.05

The number of semesters studied showed a significant effect on sleep quality, social phobia and test anxiety in a multivariate linear regression (see Table 6). University students in higher semesters reported better sleep quality and fewer symptoms of social phobia and test anxiety.

**Table 6:** Multivariate linear regression of number of semesters on mental health variables

Variable^a^	B	SE	β	Significance
PSQI—sleep quality for all students	−.062	.020	−.062	p = .002**
ESS—daytime sleepiness for all students	.000	.020	.000	p = .996
MEQ—chronotype for all students	.101	.053	.039	p = .057
PHQ-9—depression for all students	−.019	.027	−.015	p = .483
PHQ—stress for all students	.007	.018	.008	p = .688
SIAS—social phobia for all students	−.154	.059	−.054	p = .008**
PAF—test-anxiety for all students	−.075	.036	−.042	p = .038*
SWE—self-efficacy for all students	.028	.027	.021	p = .295

^a^Number of semesters was used as a predictor for PSQI, ESS, MEQ, PHQ-9, PHQ-stress, SIAS, PAF and SWE; * p < .05; ** p < .01

On the Epworth Sleepiness Scale (ESS), one-third of the students (27.2%) had a sum score higher than the cut-off of 10, indicating a high load of daytime sleepiness for both students in Germany as well as Luxembourg. 27.2% of German students showed clinically relevant daytime sleepiness, compared with 28.7% of Luxembourgish students. However, the comparison between countries was not significant (see Table 9). We found a mean of sleepiness of M = 8.33 (SD = 3.82) for the whole sample. Gender differences in daytime sleepiness were observed in the whole sample (see Table 3).

**Fig. 2:**
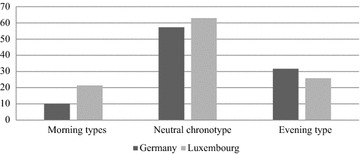
Chronotypes in percent in German and Luxembourgish students

Concerning chronotype, in the whole sample, 10.3% were morning types, 58.4% were neutral types, and 31.3% declared to be evening types (M = 46.15; SD = 9.57). Both countries had an equal proportion of subtypes (see Fig. 2; Table 8).

Chronotype differed significantly between male and female students, with men showing a later chronotype than women (see Table 3).

### Mental health

Concerning the PHQ, 25.5% of all students showed clinically elevated levels of depression (moderate depression ≥10). Of those 8.3% were above cut-off for severe symptomatology (≥15). We found mean scores for Luxembourgish as well as German students, which were mildly elevated (above cut-off for mild symptomatology). Students of both countries reported equal levels of depression and stress (see Table 9).

In relation to the diagnostic groups of the PHQ, we observed a relatively high level of students of both countries who showed a moderate or severe depressive or stress symptomatology (Table 7). Women in both countries seemed to be significantly more stressed and depressed than men (see Table 3).

**Table 7:** Proportional distribution in diagnostic groups of the PHQ-D

Symptom-atology	Depression			Stress		
	All students (%)	Germany (%)	Luxembourg (%)	All students (%)	Germany (%)	Luxembourg (%)
Minimal	30.1	30.4	27.8	45.3	45.5	41.3
Mild	37.7	37.7	37.5	37.2	37.0	38.5
Moderate	16.9	17.0	16.4	9.6	9.5	9.8
Severe	8.3	8.3	9.1	0.5	0.5	0.5

Depression and stress measured by PHQ-D, minimal <5; mild 5–9; moderate 10–14; severe >14

Social phobia was observed in 13.3% of the total sample, having a SIAS sum score higher than the cut-off of 34. Overall, students from both countries had mean scores below the cut-off score for clinical significance. Additionally, we found no significant differences between countries (for further information see Table 9). In addition, no significant differences in social phobia were detected in men and women in both countries (see Table 3).

On average, the sample had normative test-anxiety below cut-off (M = 44.59; SD = 6.59). A percentage of 9.1% of all students reported elevated test-anxiety above cut-off. No difference was found for test-anxiety between countries (see Table 9). Men and women differed significantly in their test-anxiety (Table 3).

### Physical health

Across the whole sample, 11.7% reported a chronic illness (most commonly asthma with 2.16%, hypothyroidism 1.3%, neurodermatitis 1.23%).

In both countries chronic illnesses (mental illnesses excluded) were reported, with Luxembourgish students (6.6%) reporting significantly less chronic diseases than German students (12.3%) (χ2 = 5.342; p = .010).

Concerning gender, female and male students reported an equal proportion of chronic illnesses (χ^2^ = 5.393, p = .066). In both countries, the percentage of participants reporting chronic illnesses did not differ significantly between genders.

### Self-efficacy

In summary, 15.1% of the whole sample reported scores indicating impaired self-efficacy (≤23). Self-efficacy scores did not significantly differ between countries (see Table 9), whereas there were significant sex differences with women reporting lower self-efficacy than men (Table 3).

## Discussion

Our analysis of students from Germany and Luxembourg revealed that for all variables concerning sleep quality and mental strain both countries were equally affected, which is in line with our hypotheses. *Subjective sleep quality* did not differ between German and Luxembourgish students. However, in mean the students scored above the cut-off for clinical significance concerning sleep disturbances—as measured by the PSQI [39]. Although, subscales of the Pittsburgh Sleep Quality Index did not differ between countries, a total of 42.8% of students indicated symptoms of impaired subjective sleep quality above the cut-off, and 17.9% reported symptoms of a clinically relevant sleep disorders. These prevalence rates are below previously reported findings on impaired sleep quality [6] but higher than estimates insomnia diagnosis in this population [2, 7, 8]. Nevertheless, mean daytime sleepiness (measured by ESS) was not above the cut-off. Other researchers found the highest cognitive impairments due to sleep problems and 50% experience excessive drowsiness, among university students compared to employed young adults and adolescents [40]. In the US, findings suggested that among other health related factors such as exercise, nutrition, mental health and stress management, healthy sleep habits have the highest predictive value for success in college [41]. Eden [9] concluded that excessive daytime sleepiness and low self-efficacy lead to lower academic motivation and lower academic performance.

In addition to impaired sleep quality, 18.8% reported sleep-onset latency of more than 30 min, which can be interpreted as a symptom indicating *insomnia*. In the study conducted by Taylor and colleagues [8], the relationship between insomnia and mental health problems was no longer significant after controlling for comorbid health problems even though they often co-occur. Sleep duration less than 7 h is not recommended for this age group, however, 32.6% of all students sleep less and are at risk for health problems [42].

Concerning *mental strain*, approximately 27.1% of all students suffered at least from moderate symptoms of depression. We found a slightly elevated level of depressive symptoms, and stress and signs of social anxiety for the whole sample. About 19.8% of students reported clinical relevant symptoms of social phobia, although the majority of students did not reach the cut-off for social phobia as measured by the SIAS. These prevalence rates are higher than reported by another sample of German university students, in which 14.1% fulfilled the criteria for a depressive disorder and 4.6% criteria for any anxiety disorder [18]. Even though there were no significant differences between countries in *depression, stress, social phobia* and *fear of exams*, a critical number of students seemed to be impaired in both countries. Academic success seems to be impaired by symptoms of depression as detected in the US and in a large sample of adolescents in Finland [43] and perceived stress and self-efficacy influenced academic performance [44], also these results are important for a university career. Beyond, in college freshmen, self-efficacy was a robust and consistent predictor of academic success, even more than stress. We found nearly one-quarter of our students reported clinically relevant impairments. These results demonstrate that university students have impairments in depression as well as a high stress level. Underlying mechanisms should be uncovered and implications for interventions should be developed.

As *self-efficacy* seems to be a central factor of sleep and mental health in university students it would be good to improve resources, as increase self-efficacy in university students. Various studies demonstrated that higher self-efficacy is known to be associated with less sleep problems [2] and less nightmares [3]. Self-efficacy is significantly lower in female university students than in male as reported in previous studies [45, 46].

Concerning *gender*, in the present sample female students in both countries reported significantly lower sleep quality compared to male students, which is in line with previous studies, showing lower sleep quality in female university students [2]. These findings suggest women in both countries being more vulnerable for disturbed sleep. Women tended to report more sleep disturbances and more daytime sleepiness. Chronotype was significantly different between genders, with more men reporting a slightly later chronotype, even though a later chronotype has been found to be a risk factor for disturbed sleep [47]. In addition, women tended to be more depressed, more stressed and reported more test-anxiety. These results are in line with previous findings [18, 23]. In a large global sample, Seedat and colleagues [48] found women had a higher risk for anxiety and mood disorders. Except for social phobia, these findings are in line with our sample. Interestingly, men and women report an equal number of symptoms of social phobia but this phenomenon has also been previously shown [49]. Considering these results, more attention should be paid to female students’ mental health.

The number of *semesters* studied showed a significant effect on sleep quality, social phobia, and test anxiety. Students in higher semesters reported fewer sleep problems and less anxiety. This is in contrast to previous findings which reported more mental health problems in higher semesters as health problems often co-occur with studying longer than 13 semesters [50]. However, our results are more in line with other authors, who found the highest level of sleep disturbances and stress in the first year [51].

All reported variables have an impact on subjective quality of life, which highlights the importance of further research to encounter mechanisms underlying the difference in sleep patterns in different countries. The increase in negative affect when sleep deprived [52] might lead to a vicious circle of sleep deprivation, negative affect, lower academic success, more negative interactions and lower quality of life. Further impairments including heavy drinking and physical inactivity have been concluded to be the consequence of sleep problems [53]. In addition, variables like physical activity, consummation of drugs and other health-related factors should be included in further studies. The findings in the present sample underline the importance of interventions especially designed for students.

Some *limitations* should be named. The high number of women (73.3% in the current sample; 48.0% in German universities; [54]) and very unequal sample sizes in Germany and Luxembourg might impair the generalizability of these results. Women always showed an elevated health risk and a higher stress level, so all results could have overrated the real impairments in European students [6]. Nevertheless, the current sample size is large, and all measurements were self-reports. Other than that the encountered difference in sleep disturbances between countries could be due to different cities or other factors having an impact on sleep, like stress in the specific study-subject, alcohol and drugs (Luxembourg is much closer to Holland and its legalized Marihuana) or different living environments (shared flats, student residence, living with parents). Culturally the two countries seem not very different. Furthermore, only two countries took part in this study and generalizability to other countries might be limited. The number of semesters was included in the analysis even though we had no information on degree, so the validity of this analysis is limited. More detailed analyses of different schedules in various disciplines might show impact on university students’ lives even though previous studies showed no influence on sleep quality or mental health [18, 55]. Variables worthy of consideration in further studies might be part-time or full-time studies, the number of children or jobs besides studying. Furthermore, in the present study, other comorbid health problems were not assessed even though previous research shows a strong relationship between pain and sleep [56]. Comorbid health problems might interact with mental health problems and insomnia symptoms [8].

## Conclusion

In summary, German and Luxembourgish students reported an alarming level of sleep disturbances and emotional stress with an elevated percentage on a clinical level, therefore prevention (sleep education) and specialized intervention programs are needed to enhance well-being and to prevent chronification of symptoms and impairment on academic outcomes.

## Appendix

See Tables 8 and 9.

**Table 8:** Means, standard deviations and significance level for the components of the PSQI in both countries

	Scale	Germany		Luxembourg		Significance level
		Mdn	M (SD)	Mdn	M (SD)	
(1)	Sleep quality	1		1		U = 241,562; z = −1.207; p = .228
(2)	Sleep-onset latency In minutes	2	24.47 (26.85)	2	27.84 (26.05)	U = 257,277, z = 1.888; p = .059
(3)	Sleep duration In hours	0	7.09 (1.08)	0	6.94 (1.17)	U = 256,345; z = 1.615; p = .106
(4)	Habitual sleep efficiency In percent	0	87.83 (10.11)	0	86.30 (10.92)	U = 258,207; z = 1.833; p = .067
(5)	Sleep disturbance	1		1		U = 242,547; z = −.177; p = .907
(6)	Use of sleep medication	0		0		U = 241,562; z = −.485; p = .628
(7)	Daytime dysfunction	1		2		U = 242,553; z = .473; p = .636

*Mdn* median; *M* mean; *SD* standard deviation; p ≤ 0.05

**Table 9:** Mental health differences in German and Luxembourgish university students

Variable	German university students M (SD)	Luxembourgish university students M (SD)	Significance^a^
PSQI—sleep quality	7.20 (3.66)	7.79 (4.03)	F (1, 2593) = .716; p = .389
ESS—daytime sleepiness	8.34 (3.69)	7.49 (4.26)	F (1, 2593) = .045; p = .831
MEQ—chronotype	46.11 (9.52)	47.21 (9.27)	F (1, 2593) = 1.274; p = .259
PHQ-9—depression	7.27 (4.81)	7.50 (4.79)	F (1, 2593) = .227; p = .599
PHQ—stress	5.13 (3.21)	5.19 (3.20)	F (1, 2593) = .031; p = .861
SIAS—social phobia	23.65 (10.61)	24.67 (11.10)	F (1, 2593) = 1.913; p = .167
PAF—test-anxiety	44.56 (6.57)	45.54 (6.79)	F (1, 2593) = 3.090; p = .079
SWE—self-efficacy	28.44 (4.92)	28.45 (4.62)	F (1, 2593) = .017; p = .897

^a^According to MANOVA; *M* mean; *SD* standard deviation

## Abbreviations

PSQI: Pittsburgh Sleep Quality Index
ESS: Epworth Sleepiness Scale
dMEQ: Morningness-Eveningness-Questionnaire (German version)
PHQ-D: Patient Health Questionnaire, German version
SIAS: social-interaction-anxiety scale
PAF: “Prüfungsangstfragebogen” german test anxiety questionnaire
SWE: “Selbstwirksamkeitserwartung” self-efficacy questionnaire
